# How Sustainable Is Government-Sponsored Desertification Rehabilitation in China? Behavior of Households to Changes in Environmental Policies

**DOI:** 10.1371/journal.pone.0077510

**Published:** 2013-10-31

**Authors:** Ning Liu, Lihua Zhou, J. Scott Hauger

**Affiliations:** 1 Cold and Arid Regions Environmental and Engineering Research Institute, Chinese Academy of Sciences, Lanzhou, China; 2 Asia-Pacific Center for Security Studies, Honolulu, Hawaii, United States of America; 3 China Development Bank Qinghai Branch, Xining, China; NASA Jet Propulsion Laboratory, United States of America

## Abstract

This paper undertakes a direct, comprehensive assessment of the long-term sustainability of desertification rehabilitation in China under a plausible but worst case scenario where governmental interventions, in the form of payments for environmental services (PES), will cease. The analysis is based on household behavior as well as experimental data. Our econometric results highlight the main obstacles to the sustainability of rehabilitation programs subsequent to cessation of government intervention, including specific shortfalls in households’ preference for a free ride, budget constraints, attitudes, tolerance of and responsibility for desertification, and dissatisfaction with governmental actions. We conclude that desertification rehabilitation is not sustainable in China without continued governmental intervention. The results of this study are intended to support policy makers as they consider future directions for rehabilitation sustainability.

## Introduction

Desertification is land degradation in arid, semi-arid and dry subhumid areas (Although the UNCCD definition is now formalized internationally, there has been extensive debate concerning definitions of desertification and degradation.) [1]. It affects 10–20% of all dry lands, and as much as 8% of the total land area of the world [2]. Desertification reduces the productivity of land and overburdens an ecological system, thus jeopardizing economic growth and inducing poverty [3]. To counter the significant economic, social and environmental changes that desertification brings, many attempts, generally of governmental origin, have sought to combat it on global, regional, and local levels. The most favorable outcome then is rehabilitation of the land, converting the degraded land into productive soils, especially in the agro-pastoral zone [4]–[6].

China is one of the most severely affected countries with a 385,700 km^2^ area of desertification in 2000, and 1.6 million km^2^ of susceptible arid and semiarid areas [7]. The area of desertification reached its maximum during the 1970s to the early 1980s, but has decreased continuously from the late 1980s to the present [8]. The main rehabilitation has been documented to occur from the later 1990s to the present [8]–[10].

The commonly accepted cause of desertification is the interaction of a set of natural (bio-physical) and anthropogenic factors with different temporal and spatial variability [11]. Though some argue that modern desertification in China is a product of climate change [8], an understanding that desertification has been triggered by human activities and then reversed by governmental programs has gained common acceptance and become the basis for governmental decisions [6], [12]–[19]. It is asserted that the excessive exploitation of natural resources by rural residents including overcultivation, overgrazing, deforestation, and excess irrigation predominantly worsens the process of desertification [6], [12]–[19].

The rehabilitation of desertified lands is converting the desertification areas into productive ones, especially in the agro-pastoral zone [2], [6]. Rehabilitation metrics include the size of the desertified area, soil characteristics, vegetation (grass and forest), regional environment (precipitation, sand storm, etc.), and land productivity [6], [20]. Modern rehabilitation in China since the late 1990s has been concurrent with the inception of the world’s largest and most ambitious payment for ecosystem service (PES) programs. These include the Sloping Land Conversion Program in north China, the Grazing Prohibition Program in pastoral areas, and other programs.

The Sloping Land Conversion Program (SLCP) was enacted in 2000 by the Chinese government to convert 32 million hectares of sloped land into forest land over 10 years, with a budgetary outlay of over US$30 billion and affecting 60 million households [21]. Like every PES program, SLCP has the dual goals of curtailing environmental degradation while ameliorating rural poverty. It provides participants, volunteer rural households, a compensation package including grain, cash and seedlings in exchange for reforesting and maintaining some of the sloped land that they used to cultivate for grain production [21], [22]. The compensation has time limits and it varies according to land type and forest type (economic or ecological). Though reforested land remains the property of the original land holder, tree felling is conditionally restrained.

The grazing prohibition program, begun in 2003, is another major initiative implemented in the desertified areas of north China. It possesses the same dual goals as SLCP. This 10-year program has a budget of US$3 billion and benefits 5.5 million rural households [23]. After compensation to the households, grazing is prohibited or regulated on some 90 million hectares of grassland [23]. This means that households have to adopt drylot feeding and abandon free grazing. As with SLCP, the time duration and regional variations of compensation are problematic and the implementation of the policies may not well fit local needs.

Early indications show that the environmental results of these programs in combating desertification are remarkable, although their social impacts can be controversial [24]–[30]. After a gradual period of fallowing, reforestation and grazing prohibition, soil and land productivity are recovering. Vegetation coverage and quality is increasing, the size of the desertified area is decreasing, and the incidence and extent of sand storms are also decreasing [23]. It is also documented that the main rehabilitation since the late 1990s has been concurrent with the inception of the two programs [8]–[10].

The origin, causes, impacts and processes of desertification, and the methods available to combat it in China have been discussed at great length [8]. The literature on rehabilitation, however, is limited to and focused on identifying its incidence, extension and physical dynamics (e.g. [24]–[27]). Such studies provide useful information about the physical interpretation of desertification rehabilitation, but they rarely provide insights into the social factors that promote sustainability.

In contrast to other governmental actions in environmental protection, these PES programs depend on private contracts with individual rural households, pursuing personal interests. The government is concerned about positive externalities – the improvement of the environment and social wellbeing through contracting with and compensating individual rural households. In order to achieve both the governmental goals and individual interests, contracts should impose compatible incentives. However, the interests of the two parties are not always compatible, and may even be opposite if compensation is not appropriate or after it has expired.

Earlier studies have considered only rural residents of desertified areas and have ignored the behaviors and preferences of their urban residents. People who are urban residents according to China’s special resident registration system also suffer from desertification, but they have usually been overlooked and cannot benefit directly from the poverty amelioration functions of the PES programs.

The timeliness of this sustainability study lies in the fact that the PES programs will soon be closed or modified. Thus, the questions must be asked: “How can residents of desertified areas maintain what has been achieved?” and “How can desertification be reduced in the future?” Ultimately, it is the households in the desertified areas who must directly face the problem in the long run. But what will be their decision when facing the end of governmental support? There is a timely opportunity to capture the knowledge of those households and their experience of the initial programs to date.

This paper therefore undertakes a comprehensive assessment of sustainability, based on household behavior, intentions and preferences under a plausible but worst case post-PES scenario: where the PES program will be terminated. The purpose is to identify the likely impacts of PES program termination on household behaviors and thus on the longer-term sustainability of rehabilitation of desertification. The analysis is based on data obtained from a carefully designed survey that was conducted in a typical agro-pastoral zone in China. Both household and village level data were collected via in-person interviews with randomly selected households and with village leaders.

## Materials and Methods

### Theoretical Models

Household behaviors may be explicated in the context of the following closed models: Desertification rehabilitation (**Q**) is a function of governmental intervention **S**, household *i’*s response to the intervention **R_i_(S)**, and natural factors **N** as follows [31]:
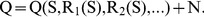
(1)


Variable **N** can be eliminated if considering exogeneity and the short time extent. Then introducing equation (1) into the household indirect utility function 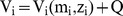, where **m_i_** and **z_i_** are a household’s income and other attributes, there is a new indirect utility function with the transformation:
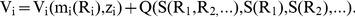
(2)


A two-stage analysis is then employed in the light of Choice Experiment theory (CE), where stage I indicates a worse condition of desertification, and stage II indicates a better rehabilitation condition. A clarification is that rehabilitation is not yet completely accomplished at stage II. The model supposes that a household will pay **W** for the rehabilitation attempt **S**, only if the following equation (5) is satisfied.
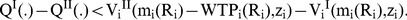
(3)


This CE model is used to investigate individual preference towards different product packages on the basis of the economic theories of Utility Maximization and Value. Originally used for individual behavior studies in selecting commercial products [32]–[33], it was gradually introduced into research on environmental and natural resources [34]–[36], and then adapted to be a yardstick to evaluate individual behaviors in the field of environment, health and other public goods [37]–[38].

For the experiment at hand, we adopt WTP (willingness to pay) and WTA (willingness to accept) to be proxies of household behaviors towards environmental protection. WTP can be defined as the maximum price which the household will be willing to pay for further desertification rehabilitation and WTA as the minimum price at which the household wishes to give up further rehabilitation. Specifically, WTA represents a pecuniary preferred compensation for the difference of PES programs’ performance on rehabilitation and individual expectation. The pecuniary number that households give or accept is the result of behaviors with rational preference and decision. WTP and WTA are functions of a household’s behaviors as follows:
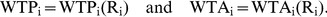
(4)


The disparity between WTP and WTA is an additional measure that can provide insight to household behavior. It has been theoretically and empirically documented, and is generally larger for non-market goods and smaller for ordinary goods [37]–[38]. The literature provides several explanations for this. One is based on the concept of reference-dependent preferences [39] where initial endowment and changes in reference point lead to changes in preference. The second is the availability or unavailability of substitutes for non-market products [40]. The third attributes disparities to deficiencies in study design [37], [41], and the fourth considers the ways in which uncertainty, irreversibility and learning opportunities influence a person’s choice [42]. By considering these stated biases, an investigation into the factors that affect WTA/WTP disparity in the case of desertification rehabilitation services can provide a significant resource to launch a new policy or to properly guide and implement the already established one [43].

### Study Area

The study area, Yanchi (*E106°30*′*–107°47*′, *N37°4*′*–38°10*′), is a county of Ningxia province located in the arid and semiarid region of northwest China. Geographically, it is a typical transition zone between the Mu Us Desert and the Loess Plateau with a climate that varies from semiarid to arid, and vegetation that varies from steppes to desert steppes. Annual precipitation is 250–350 mm, much less than the annual evaporation of approximately 2,900 mm [44]. Local economic activities mainly rely on natural resources, the land output, grassland and several minerals, with GDP per capita being 70% of the national average in 2010. Eighty per cent of the population is agrarian and their contribution to the local GDP is only 15%, of which 39% is from crop cultivation and 45% is from animal husbandry [44].

Historically, the area was known for grazing land and animal husbandry, but during recent decades, it has been deteriorating, mainly due to desertification. According to some studies, desertified land in the area covered as much as 3,611 km^2^, accounting for 52% of the total acreage [45]. To combat desertification, local governments have made serious attempts, among which the most important are the Sloping Land Conversion Program (SLCP) in 2001, and a grazing prohibition project, beginning in 2002(Time of launch of PES programs is varied by region.). Those policies and projects have been considered to be successful, and it is stated widely that local desertification rehabilitation has been observed and tangibly achieved after their implementation [46].

However, the designed lifetime of these policies and projects will soon expire. The future sustainability of rehabilitation will become problematic if there are no follow-up policies and projects. In order to support decision making regarding the future sustainability of desertification rehabilitation, there is a need to better understand the impacts of these programs over the last decade. Moreover, because only rural residents (farmers and pastoralists) were compensated for their direct contributions to environmental protection, there is a need to consider the reactions of other residents (town and city). The results of this study are intended to support policy makers as they consider future directions for rehabilitation sustainability.

### Data

The sustainability of desertification rehabilitation is analyzed under the plausible scenario that after the end of government programs, residents themselves will contribute to continued rehabilitation attempts without external support. As mentioned, both the rural and urban residents in the desertification area are troubled by this environment, but the rural residents suffer more because their living is directly restrained by desertification. While PES programs offer rural residents both an improved environment and monetary compensation, urban residents benefit from the improved environment, but receive no compensation. In our hypothesis, stopping of PES programs pushes both rural and urban residents to face a dilemma of whether or not to continue to fund desertification rehabilitation in the absence of government support.

During data collection, respondents were first asked if they were satisfied with the results of current PES programs (desertification rehabilitation or improvement of local desertification status) and if they were personally willing to sponsor a new, similar program. Those who were less than satisfied were then asked to select a pecuniary preferred compensation for the difference between the perceived success of current PES programs and their individual expectation, which is defined as WTA. Then respondents who agreed to pay for a new program of desertification rehabilitation were requested to select from a range of options their preferred pecuniary contribution toward future rehabilitation. This is defined as WTP.

Two groups of households were interviewed under a stratified random sampling strategy covering 8 townships from the north to the south (The sample is geographically stratified from North to Mid to South, town by town, to indicate the severity of desertification and from the rich to the poor to sample across all economic strata within the county.). Each group was comprised of households both living in the rural region and living in town (Usually, only the income of rural households would be directly affected by desertification, though it degrades the environment for both urban and rural residents). One group was queried regarding a range of numeric pecuniary contribution options for WTP and WTA (experiment V_a_ and V_d_). This group was also offered an open choice of numeric pecuniary WTP (experiment V_b_), to investigate the embedding effect, reliability and internal consistency of the different results from experiment V_a_. The other group was only asked to make a binary choice for the acceptance or rejection of randomly designated pecuniary contribution options (experiment V_c_).

The pooled samples were 447 for experiment V_a_ and experiment V_b_, 165 for experiment V_c,_ and 169 for experiment V_d_, respectively. However, the sample pool for disparity analysis of WTA and WTP was 131, since only those who decided to contribute can be counted. The survey also reported information on households’ social economics, demography, and perceptions on desertification and rehabilitation.

### Empirical Models

To investigate for an embedding effect, reliability and internal consistency of the different elicitations, the hypothesis proposed by Christie (2001) [47] was first tested as:
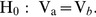
(5)


The null hypothesis (H_0_) is that there is no significant difference in the two elicitation treatments. A priori it is expected that the null hypothesis will not be rejected.

Disparity of WTA and WTP are measured by the ratio of WTA/WTP. The ratio adopted by Horowitz and McConnell (2002) [37] was mean WTA/mean WTP, while we directly use the asserted value. Aggregation of household and groups WTA/WTP [37] are also compared by,
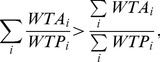
(6)where *i* represents the household and values for the ratio are obtained from experiment V_a_, V_b_ and V_d_ respectively.

To test the effects of household behaviors on desertification rehabilitation, an estimation strategy for experiments V_a_, V_b_ and V_d_ is represented by:
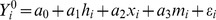
(7)where *i* indicates the investigated household, *h* is a vector of demographic variables, *x* denotes a vector of impact factors such as profession, income, severity of desertification and the interaction of profession and severity of desertification, and *m* is a vector of perception of desertification rehabilitation including Q_1_, Q_2_ and Q_3_ (Q_1_: Do you feel some improvement of the natural environment in your county relative to previous years? Q_2_: Which is the main reason in your opinion about the desertification in your county? Q_3_: Do you think that implementation of ecological protection policies plays an active role for the environment or not in your county? The response to Q_1_ and Q_3_ is a binary option, Yes or No, and the answers to Q_2_ are governmental actions, individual behaviors, natural effect and others.). It is necessary to state that (1) the robustness of variables is highly dependent on data availability and the model’s significance; and (2) severity of desertification cannot be numerically characterized at this time. Because the sample is geographically stratified north to south, we can use different towns as a proxy for that variable. 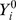 is the outcome of interest for investigated households’ *WTP* and *WTA* in experiments V_a_, V_b_ and V_d_. The parameters of interest in model (7) are *α_1_*, *α_2_* and *α_3_*, which show the effects of household actions on desertification rehabilitation. 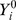 is a final decision measure, such as positive, negative, indifference or decision to be a free rider.

To test if a given household acts similarly under a yes/no dichotomous choice condition, the estimate model for experiment V_c_ is proposed as:
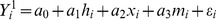
(8)where 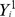 is a binary variable to indicate household *i*’s response (yes = 1, no = 0) for a randomly assigned value of WTP. Definitions of other variables remain the same as model (7). The interaction of profession and severity of desertification is employed only in this model.

The model that was used to estimate what will impact the disparity of WTA and WTP is:
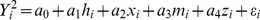
(9)where 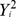 is a dependent variable *ratio_i_* (*i* = 1 and 2 for V_a_ and V_b_ respectively), and *z_i_* is a vector of the other documented controls to explain the disparity including income effect, initial endowment effect, substitution, study design and uncertainty.

Income effect is presented as annual income. Initial endowment will be represented by a dummy variable in comparison of household income between 2011 and 2001 when the main interventions were launched. Because it is impossible to identify the exact household endowment in 2001, we use the dummy variable to roughly distinguish people whose income has been reduced by desertification or not. That means that the initial endowment is 1 if household income is more in 2010 than in 2001, and 0 if it is less.

Any substitution effect will be excluded since desertification and its rehabilitation are pure public goods without substitutes. Because many researchers have argued that the effect of study design is not so clear (e.g. [37]–[38], [48]), it will be deliberately ignored in our study. It is plausible, in our closed model, to use age as a proxy for uncertainty and knowledge of desertification issues because most residents of the study area have lived there since birth, while the desertification process has lasted more than 100 years.

## Results and Discussion

### Descriptive Summaries

Table 1 summarizes the descriptive statistics of this study. The rate of respondents’ choice to contribute to future remediation attempts is not much varied among the four experiments. Choice acceptance differed between the specified pecuniary contribution test (V_a_) and the open choice test (V_b_), rejecting the null hypothesis (equation 5) (P<0.0001), and ratio_1_ is found to be less than ratio_2_. This result is theoretically ambiguous but not novel in practice. Incomes are unstable and future income may be discounted for residents with jobs like farming, herding or part time employment. Even residents with permanent jobs, like government employees, are subject to currency inflation. Although reducing present consumption may be a more rational choice, residents prefer to promise a higher total payment over a long term installment rather than to make a smaller, lump-sum payment from current disposable income. Correspondingly, ratio_1_ is less than ratio_2_.

**Table 1:** Summary statistics.

Variables	V_a_	V_b_	V_c_	V_d_	Ratio_1_	Ratio_2_
**N (sample** **size)**	447	447	165	169	131	131
**Choice** **acceptance**	0.54	0.54	0.64	0.60		
**Difference**	V_a_>V_b_ (P<0.0001)				***∑WTA/∑WTP***	
					1.24	1.97
					***∑(WTA/WTP)***	
					381.95	604.25

Though the hypothesis in equation (5) fails to pass the test, the hypothesis in equation (6) is well demonstrated. This indicates that our result is concordant with common empirical results of the disparity of choice between groups and households [28].

### Household’s Behaviors among Group I

The Ordinary Least Squares (OLS) regression results of experiment V_a_, experiment V_b_ and experiment V_d_ are given in Table 2. Panel A shows demographic information, Panel B shows impact factors, and Panel C shows perceptions of desertification rehabilitation based on three questions to the participants (Q_1–3_). Gender, profession and income are of statistical significance for experiment V_a_, whereas age, profession and income are of statistical significance for experiment V_b_. The interaction of profession and severity of desertification is considered but finally rejected as less than significant. The perception represented by Q_3_ is also rejected in experiment V_a_ and experiment V_b_ for a similar reason.

**Table 2:** Regressions of V_a_, V_b_ and V_d_.

Independent Variable	LogWTP(V_a_)	LogWTP(V_b_)	LogWTA(V_d_)
**Panel A**			
** Sex**	0.4622***(0.1558)	0.2619(0.1614)	
** Resident category** a	−0.2372(0.1981)	−0.0874(0.2051)	
** Age**	−0.0102(0.0069)	−0.0163**(0.0072)	
** Education**	−0.0122(0.0204)	−0.0190(0.0212)	
** Family size**	0.05144(0.0562)	0.04374(0.0577)	
**Panel B**			
** Desertification** b	−0.0108(0.2074)	−0.2786(0.2148)	0.8362**(0.3803)
** Profession**	−0.7312***(0.2120)	−0.4646**(0.2194)	
** Log(income)**	0.5305***(0.1285)	0.4713***(0.1329)	
**Panel C** c			
** Q**_1_	0.0615(0.0649)	0.07469(0.0673)	
** Q**_2_	−0.0538(0.2477)	−0.2411(0.1544)	
** Q**_3_			1.4396**(0.6244)
**Intercept**	−0.6732(1.3327)	0.1632(1.3805)	3.8878***(0.6100)
**N**	447	447	165
**F Value**	3.97***	2.69***	2.23**
**R-Square**	0.1396	0.0984	0.0606

a Residents in China are legally separated into urban and rural residents.

b Severity of desertification.

c Q_1_: Do you feel some improvement of the natural environment in your county relative to previous years? Q_2_: Which is the main reason in your opinion about the desertification in your county? Q_3_: Do you think that implementation of ecological protection policies plays an active role for the environment or not in your county?

d ** and *** indicate the significance at 5% and 1% level respectively.

Specifically, as indicated by the value of log(WTP), men’s willingness to contribute to future remediation efforts is significantly higher (46.22%) than for women in experiment V_a_. Willingness to contribute is significantly less by 1.63% as age increases in experiment V_b_.

Professions are roughly classified into a) those which are directly affected by desertification and with unstable income (e.g. farmers), and b) those which are not directly impacted by desertification, with or without out stable income (e.g. governmental employees and self-employed businessmen). Log(WTP) of those in occupation a) is significantly higher by 73.12% and 46.46% than in occupation b) in experiment V_a_ and V_b_, respectively. It is also found that the log(income) will significantly augment the log(WTP), at 53.15% and 47.13% for both experiment V_a_ and experiment V_b_.

Income’s effect on WTP has been widely documented in other studies [37]–[38]. The professional disparity is also not of much novelty. Farmers and those in related professions suffer more from desertification. They are the most vulnerable group. Survival will be more important in the short run given the poverty of rural households in the study area. A possible explanation of the observed higher log(WTP) for males and lower log(WTP) for elders may be the power to decide household expenditure distribution. A higher awareness of environmental issues for men, and a higher tolerance of the degraded environment for the elders also appear to be factors. Under the historic and cultural traditions of the study area, men usually receive more education and get more experience with the outside world than women. They are typically in charge of the family’s finances.

To get the model of significance we used a backward strategy in WTA regression (***V_d_***). We find that more suffering from desertification will increase log(WTA) up to 83.62%; and the less the praise of governmental rehabilitation programs, the more compensation will be required, with an increase of log(WTA) of 0.16%. Once again, due to the lack of more clear measures of the impact of desertification in the study area, the geographic location of different towns was used to represent the severity level.

### Household’s Behaviors among Group II

Table 3 presents regression results for experiment V_c_. A Linear Probability Model (LPM) estimate and then a logit estimate are presented. Panel A, B and C represent the same variables as above. Both estimates show a significant positive relation of gender, income and Q_1_, and a significant negative relation of profession with the dependent variable. We also looked at the interaction between profession and severity of desertification (*Desert×prof*) here, which shows a significant positive relation with the dependent variable.

**Table 3:** Regression of V_c_.

Independent Variables	Logit	LPM
**Panel A**		
** Sex**	1.9959*** (0.6665)	−0.2254*** (0.0765)
** Resident category** a	−0.5927(0.8590)	0.0833(0.1033)
** Age**	0.0013(0.0273)	−0.0009(0.0033)
** Education**	0.0443(0.0835)	−0.0014(0.0108)
** Family size**	0.0598(0.2688)	−0.0041(0.0322)
**Panel B**		
** Desertification** b	−0.6649(1.1988)	0.1595(0.1240)
** Profession**	−1.8568*(1.1988)	−0.2739**(0.1146)
** Desert×prof** c	1.0868*(0.5872)	−0.1748***(0.0635)
** Log(income)**	−0.2906**(0.4302)	0.0632**(0.0314)
**Panel C** d		
** Q_1_**	−0.4339**(0.2104)	0.0581*(0.0305)
** Q_2_**	0.5835(0.3919)	−0.0641(0.0512)
**Intercept**	1.0790(5.4077)	
**N**	165	165
	−**2 Log L** = 130.99; **Likelihood Ratio** = 28.89***	**R-Square = **0.84
	**Score = **26.33***; **Wald = **19.48*	F = 58.40***

a Residents in China are legally separated into urban and rural residents.

b Severity of desertification.

c Interaction of profession and severity of desertification.

d Q_1_: Do you feel some improvement of the natural environment in your county relative to previous years? Q_2_: Which is the main reason in your opinion about the desertification in your county? **^e^** *, ** and *** indicate the significance at 10%, 5% and 1% level respectively.

In detail, the probability for men to accept the random assigned WTP is 22.54% less than women. One whose profession is type a) also exhibits a high likelihood to accept (27.39%), and higher income will increase the probability of acceptance up to 6.32%. But the interaction of profession and severity of desertification shows that as desertification severity increases, the probability for residents holding job type a) to accept the random assigned WTP bid increases by 17.48%. In addition, awareness of environmental improvement significantly also increases the probability of acceptance (5.81%). Here only the LPM results are presented. Results from the logit model seem not so robust.

These results are in accord with results of experiment V_a_ and experiment V_b_,though the dependent variable here is probability to accept a randomly assigned WTP. The introduction of the interaction (Desert×prof) indicates that the severity of desertification somehow reverses the impact of profession on the probability to accept a randomly assigned WTP. What should be emphasized is that the results in this analysis address the probability to accept a randomly assigned value, but are not a measure of the desire of the resident to pay. So it is possible that when the residents of the more seriously degraded regions express a lower probability to accept the value, it may be that the given value is too large for them to afford.

### Disparity between WTA and WTP

Ordinary least squares (OLS) results of ratio_1_ and ratio_2_ are given in table 4. Panel A represents demographic information. Panel B indicates impact factors. Panel C indicates perceptions of desertification rehabilitation, and Panel D explores explanations of the observed disparity of WTA and WTP. Although the severity of desertification is significant in our analysis of WTA, we exclude it and its interaction with profession from the analysis here due to sample limitations. Income and age are both assigned to panel D to indicate the effects of income and learning. Q_3_ is not significant in experiments V_a_ and V_b_, but is significant in experiment V_d_, so it is included here.

**Table 4:** Regression of Ratio_1_ and Ratio_2_.

Independent Variables	Ratio_1_	Ratio_2_
**Panel A**		
** Sex**	1.4687(1.9906)	1.8456(2.6106)
** Resident category** a	2.5652(2.4909 )	−1.6163(3.2667)
** Education**	−0.0632(0.2180)	0.2702(0.2859)
** Family size**	0.6218(0.8870)	0.0156(1.1633)
**Panel B**		
** Profession**	−5.5299*(2.9452)	−8.0659**(3.8625)
**Panel C** b		
** Q_1_**	−2.2103*(1.2994)	−1.0251(1.7041)
** Q_2_**	4.0472*(2.1290)	4.5043(2.7920)
** Q_3_**	0.0327(0.9332)	−1.7788(1.2238)
**Panel D**		
** Endowment**	3.2435(3.2647)	5.5974(4.2815)
** Log(income)**	−7.4634***(2.5867)	−8.0664**(3.3923)
** Age (learning experience)**	0.1738**(0.0835)	0.2807**(0.1094)
**Intercept**	66.6814(23.4840)	73.7005(30.7980)
**N**	169	169
**F value**	2.19**	1.96**
**R-Square**	0.1956	0.0874

a Residents in China are legally separated into urban and rural residents.

b Q_1_: Do you feel some improvement of the natural environment in your county relative to previous years? Q_2_: Which is the main reason in your opinion about the desertification in your county? Q_3_: Do you think that implementation of ecological protection policies plays an active role for the environment or not in your county?

c *, ** and *** indicate the significance at 10%, 5% and 1% level respectively.

Profession type b) significantly narrows the disparity, with values of 5.529 and 8.0658 for Ratio_1_ and Ratio_2_ respectively. It has the same effect as income which also decreases the two ratios with values of 7.4634 and 8.0644. Using age as a proxy for learning experience of the value of desertification rehabilitation, it is found that the higher the age the higher the disparity of WTA and WTP. The disparity is 0.1738 and 0.2708 for ratio_1_ and ratio_2_. Q_1_ and Q_2_ showed significant effects for ratio_1_ respectively at 2.2103 and 4.0472, but not for ratio_2_.

### Negative Choice of WTA and WTP

We also investigated the reasons that some residents choose not to contribute (see Table 5). Nearly half of this group asserts that they are too poor to afford a contribution for rehabilitation, which is consistent with our finding of the impact of income on WTP. Moreover, more than 40% of respondents can be categorized as free riders. They prefer to transfer the cost of environmental improvement to government. Even so, we found that residents are aware of the significance of environmental problems. For example, even though they are not willing to pay, only a few indicate that they are indifferent to this problem. Also, some do not believe that desertification rehabilitation policies and programs will reach the desired result.

**Table 5:** Reasons for negative response for WTP.

Reason^1^	V_a_/V_b_	V_c_
Affordability	0.49	0.50
Indifference	0.03	0.02
Governmental duty	0.43	0.40
Unexpected results	0.05	0.08
N	119	50

1 Affordability means the residents assert they cannot afford the contribution for further reversal. Indifference means the residents are indifferent about desertification reversal. Governmental duty indicates the residents think that it is the government’s duty to combat desertification. Unexpected results mean that the residents think that even if they contribute, the outcome will be less than their expectation.

## Conclusion

China has suffered from desertification for many years [7]. But in recent years the government has adopted some ambitious environmental policies to combat and then rehabilitate desertification. Although desertification is understood to result from an interaction of both natural and anthropogenic factors [11], desertification rehabilitation in China is directed at altering human behavior in a continuing process of institutional intervention [6], [12]–[19]. Historically, the primary rehabilitation attempts have occurred since the late 1990s [8]–[10], when national PES programs, such as the Sloping Land Conversion Program and grazing prohibition programs, were implemented. However, these programs have time limits, and, the looming challenge is what may happen after termination of government sponsored PES programs.

Our analysis provides a comprehensive, *ex ante* assessment of the long run sustainability of desertification rehabilitation in China by examining household behavior under a plausible but worst case scenario where the PES programs will be terminated. As a result of the analysis, the following conclusions can be cautiously drawn: (1) Income is still the most constraining factor for households to contribute funds for continuing desertification rehabilitation in the absence of government support. (2) Residents whose profession can be directly impacted by desertification hold a stronger willingness to ameliorate the problem. (3) Variations due to the local severity of desertification are not conspicuous. (4) Households seem indifferent to the causes of desertification and ignorant of the governmental contribution to desertification rehabilitation, though sometimes they praise the improvement of environmental quality. (5) Young persons and males tend to value environmental remediation higher than older persons and females. (6) Respondents are not well satisfied with the accomplishments of governmental actions to date.

We argue that rehabilitation cannot be sustained by relying on household contributions without governmental input, because we found that households prefer to free ride to some extent, and because they are usually constrained by household budget. The evidence also indicates that (1) Those whose profession is not directly impacted by desertification are richer but have less concern for environmental improvement; (2) The younger generation and males care most about environmental problems. But they tend to emigrate to the towns, and thereby reduce their passion to combat desertification in the rural regions; (3) Although the endemic desertification is derived in part from human contributions, individual households do not take into account the impact of their behavioral decisions on environmental protection; and remarkably, (4) Residents are not well satisfied with accomplished governmental actions to date.

Our study has some limitations. Without panel data, we used cross-sectional data 10 years after the PESs’ implementation. Due to differences in sampling strategy and site selection, some of our findings may differ from others. But our study site is a traditional transitional zone in geography, climate and economic activity, which implies that our analysis may be more broadly applicable.

## Supporting Information

File S1 The Questionnaires used in this study.
(DOC)
