# Supplementary material for: How Sustainable Is Government-Sponsored Desertification Rehabilitation in China? Behavior of Households to Changes in Environmental Policies
Source: PLoS One. 2013 Oct 31;8(10):e77510. doi: 10.1371/journal.pone.0077510 (PMC3815025; doi:10.1371/journal.pone.0077510)
Supplement: File S1 — The Questionnaires used in this study. (DOC) [file pone.0077510.s001.doc]

**S1: Questionnaires**

**This data is private confidential and cannot be released or used without a permission, according to Section 3(14) of the Statistic Law of the People’s Republic of China.**

**Investigation on Environmental Protection Policies and Household’s Behavior[[1]](#footnote-2) A**

Subject No.: Name of Subject：

The household is：(1) Native (2) Nonnative (Time to immigrate in )

Address： County/District Town/Street  Village/Community No.

Name of Investigator:

Date:

**Please read the following information to the subject before beginning of your investigation：**

Hello! We are a research team of the Chinese Academy of Sciences. We invite you to join our research investigation towards environmental protection policies (the Sloping Land Conversion Program in north China, and the grazing prohibition program in pastoral area) and household’s behavior. To be an understanding of desertification and its combat at your hometown, we may ask you some information including your family, livelihood, production and your attitude towards desertification and measures to combat it. Your participation is voluntary but will be highly appreciated. Your information will be confidentially protected and only used for academic research and you can cancel the interview at any time for any reason if you are unwilling to join in.

The interview will be about 30 minutes. You will not be paid but will be given a small gift as an expression of our thanks for your support and cooperation.

Once again we promise that the information collected will be highly confidential and can only be accessed by researchers. All your responses will not be related to your identity.

Thank you again for your cooperation!

1. **General information**
   1. Nationality ______
2. Han b) Others
   1. Gender ______
3. Male b) Female
   1. Age _________(Real age in number)
   2. Resident category ______
4. Rural b) Urban
   1. Education level ______ (Years of school attendance)
   2. Profession
5. Crop farming b) Animal husbandry c) Other off-farm labor d) Private businessman e) Governmental employee

f) Others

- 1. Family size ______ (Number of persons who are legally registered in your family.)
  2. How many of family members are off-farm labors?

1. 1 b) 2 c) 3 d) 4 e) 5 f) More____(detail number)
   1. How much is the annual income of your family in last year? ______ (In Chinese RMB.)
   2. Are you and your family satisfied with this annual income (in last year)?
2. Great satisfaction b) Fair c) No
   1. Does annual income of your family vary in the past several years?
3. Great increase b) A bit increase c) No change d) A bit decrease e) Great decrease
4. **Investigation on local desertification and PES programs**
   1. Do you think the local natural environment has improved compared with 5 year ago or ten years ago?
5. Great improvement b) A bit improvement c) No change d) A bit deterioration e) Great deterioration
   1. What are the major reasons of local desertification in your opinion? (Your choices could be more than one option.)

a) Mismanagement of government b) Over-cultivation c) Land fallow d) Over-exploitation of underground water e) Overgrazing

f) Deforestation g) Climate change or other natural factors h. Others

- 1. Is the Sloping Land Conversion Program implemented in your home town?
  2. Yes b) No
  3. If yes for 2.3, when was the Sloping Land Conversion Program implemented in your home town? _________
  4. Is your family involved in the Sloping Land Conversion Program?

1. Yes b) No
   1. If yes for 2.5, when was your family involved in the Sloping Land Conversion Program? _________
   2. If yes for 2.5, how much compensation in money you family has get in last year from the Sloping Land Conversion Program? _________
   3. Do you think the Sloping Land Conversion Program has positive effect on environment?
2. Very positive b) Positive c) Fair d) Little e) No
   1. Is the Grazing Prohibition Program implemented in your home town?
      - - 1. Yes b) No
   2. If yes for 2.9, when was the Grazing Prohibition Program implemented in your home town? _________
   3. Is your family involved in the Grazing Prohibition Program?
3. Yes b) No
   1. If yes for 2.11, when was your family involved in the Grazing Prohibition Program? _________
   2. If yes for 2.11, how much compensation in money you family has get in last year from the Grazing Prohibition Program? _________
   3. Do you think the Grazing Prohibition Program has positive effect on environment?
4. Very positive b) Positive c) Fair d) Little e) No
   1. Overall, are you and your family satisfied with the efforts made by government to combat desertification?
5. Great satisfaction b) Fair c) No d) Never thought of that
6. **Investigation on household behaviors towards environmental protection (desertification rehabilitation)**
   1. If the current PES programs (the Sloping Land Conversion Program and the grazing prohibition program) stop in the near future, would your family mind paying for a further improvement of local desertification by yourself? (It means a project to combat desertification may need support from local households and will only be supported by local households in the future.)

Yes b) No

- 1. If yes for 3.1: There will be a project lasting for 5 years, how much would your family pay for it every year in Chinese RMB? (Your family contribution for this project will be collected yearly for 5 years.)

1, 3, 5, 10, 15, 20, 30, 50, 80, 100, 150, 200, 500, more than 500 _________(Please give the exact number if your choice is more than 500)

- 1. If yes for 3.1: If contribution from your family to the project mentioned in 3.2 will be collected in the beginning of the year, how much would your family pay for it? (It also means your family contribution for this 5 years project will be collected one time.) _________ (Please give the exact number of money in Chinese RMB.)
  2. If no for 3.1, what is your reason?

1. My family cannot afford the contribution for further reversal.
2. My family is indifferent about desertification reversal.
3. It is the government’s duty to combat desertification.
4. Even if we contribute, the outcome will be less than their expectation.
5. Others
   1. Does the local combat to desertification reach your expectation since the launch of PES programs (the Sloping Land Conversion Program and the grazing prohibition program)?
6. Yes b) No
   1. If no for 3.5, how much you may want to be compensated by the government yearly for a 5 years proposed range?

1, 3, 5, 10, 15, 20, 30, 50, 80, 100, 150, 200, 500, more than 500 _________(Please give the exact number if your choice is more than 500)

1. **Reliability**
   1. At last, we thank you so much for your cooperation and we want to know if you are sure about all the answers you have given above.

a) Very affirmative b) Affirmative c) Not affirmative

**Thanks again for your cooperation!**

**This data is private confidential and cannot be released or used without a permission, according to Section 3(14) of the Statistic Law of the People’s Republic of China.**

**Investigation on Environmental Protection Policies and Household’s Behavior[[2]](#footnote-3) B**

Subject No.: Name of Subject：

The household is：(1) Native (2) Nonnative (Time to immigrate in )

Address： County/District Town/Street Village/Community No.

Name of Investigator:

Date:

**Please read the following information to the subject before beginning of your investigation：**

Hello! We are a research team of the Chinese Academy of Sciences. We invite you to join our research investigation towards environmental protection policies (the Sloping Land Conversion Program in north China, and the grazing prohibition program in pastoral area) and household’s behavior. To be an understanding of desertification and its combat at your hometown, we may ask you some information including your family, livelihood, production and your attitude towards desertification and measures to combat it. Your participation is voluntary but will be highly appreciated. Your information will be confidentially protected and only used for academic research and you can cancel the interview at any time for any reason if you are willing to join in.

The interview will be about 30 minutes. You will not be paid but will be given a small gift as an expression of our thanks for your support and cooperation.

Once again we promise that the information collected will be highly confidential and can only be accessed by researchers. All your responses will not be related to your identity.

Thank you again for your cooperation!

1. **General information**
   1. Nationality ______
2. Han b) Others
   1. Gender ______
3. Male b) Female
   1. Age _________(Real age in number)
   2. Resident category ______
4. Rural b) Urban
   1. Education level ______ (Years of school attendance)
   2. Profession
5. Crop farming b) Animal husbandry c) Other off-farm labor d) Private businessman e) Governmental employee

f) Others

- 1. Family size ______ (Number of persons who are legally registered in your family.)
  2. How many of family members are off-farm labors?

1. 1 b) 2 c) 3 d) 4 e) 5 f) More____(detail number)
   1. How much is the annual income of your family in last year? ______ (In Chinese RMB.)
   2. Are you and your family satisfied with this annual income (in last year)?
2. Great satisfaction b) Fair c) No
   1. Does annual income of your family vary in the past several years?
3. Great increase b) A bit increase c) No change d) A bit decrease e) Great decrease
4. **Investigation on local desertification and PES programs**
   1. Do you think the local natural environment has improved compared with 5 year ago or ten years ago?
5. Great improvement b) A bit improvement c) No change d) A bit deterioration e) Great deterioration
   1. What are the major reasons of local desertification in your opinion? (Your choices could be more than one option.)

a) Mismanagement of government b) Over-cultivation c) Land fallow d) Over-exploitation of underground water e) Overgrazing

f) Deforestation g) Climate change or other natural factors h. Others

- 1. Is the Sloping Land Conversion Program implemented in your home town?
  2. Yes b) No
  3. If yes for 2.3, when was the Sloping Land Conversion Program implemented in your home town? _________
  4. Is your family involved in the Sloping Land Conversion Program?

1. Yes b) No
   1. If yes for 2.5, when was your family involved in the Sloping Land Conversion Program? _________
   2. If yes for 2.5, how much compensation in money you family has get in last year from the Sloping Land Conversion Program? _________
   3. Do you think the Sloping Land Conversion Program has positive effect on environment?
2. Very positive b) Positive c) Fair d) Little e) No
   1. Is the Grazing Prohibition Program implemented in your home town?
      - - 1. Yes b) No
   2. If yes for 2.9, when was the Grazing Prohibition Program implemented in your home town? _________
   3. Is your family involved in the Grazing Prohibition Program?
3. Yes b) No
   1. If yes for 2.11, when was your family involved in the Grazing Prohibition Program? _________
   2. If yes for 2.11, how much compensation in money you family has get in last year from the Grazing Prohibition Program? _________
   3. Do you think the Grazing Prohibition Program has positive effect on environment?
4. Very positive b) Positive c) Fair d) Little e) No
   1. Overall, are you and your family satisfied with the efforts made by government to combat desertification?
5. Great satisfaction b) Fair c) No d) Never thought of that
6. **Investigation on household behaviors towards environmental protection (desertification rehabilitation)**
   1. If the current PES programs (the Sloping Land Conversion Program and the grazing prohibition program) stop in the near future, would your family mind paying for a further improvement of local desertification by yourself? (It means a project to combat desertification may need support from local households and will only be supported by local households in the future.)

Yes b) No

- 1. If yes for 3.1: There will be a project lasting for 5 years, would your family mind paying for it by the following given amount of money in Chinese RMB every year? (Your family contribution for this project will be collected yearly for 5 years.)

(We will randomly give subject a number from the range of 1, 3, 5, 10, 15, 20, 30, 50, 80, 100, 150, 200, 500, more than 500 according to statistical skill).

1. Yes b) No
   1. If no for 3.1, what is your reason?
2. My family cannot afford the contribution for further reversal.
3. My family is indifferent about desertification reversal.
4. It is the government’s duty to combat desertification.
5. Even if we contribute, the outcome will be less than their expectation.
6. Others
7. **Reliability**
   1. At last, we thank you so much for your cooperation and we want to know if you are sure about all the answers you have given above.

a) Very affirmative b) Affirmative c) Not affirmative

**Thanks again for your cooperation!**

1. Policies here mentioned are (PES) programs, the Sloping Land Conversion Program in north China, and the grazing prohibition program in pastoral areas. [↑](#footnote-ref-2)
2. Policies here mentioned are (PES) programs, the Sloping Land Conversion Program in north China, and the grazing prohibition program in pastoral areas. [↑](#footnote-ref-3)
